# Chromosomal copy number alterations for associations of ductal carcinoma *in situ* with invasive breast cancer

**DOI:** 10.1186/s13058-015-0623-y

**Published:** 2015-08-13

**Authors:** Anosheh Afghahi, Erna Forgó, Aya A. Mitani, Manisha Desai, Sushama Varma, Tina Seto, Joseph Rigdon, Kristin C. Jensen, Megan L. Troxell, Scarlett Lin Gomez, Amar K. Das, Andrew H. Beck, Allison W. Kurian, Robert B. West

**Affiliations:** Department of Medicine, Stanford University School of Medicine, 291 Campus Drive, Stanford, CA 94305 USA; Department of Pathology, Stanford University School of Medicine, 300 Pasteur Drive, Stanford, CA 94305 USA; Pathology and Laboratory Medicine, Palo Alto Veterans Affairs Health Care System, 795 Willow Road, Palo Alto, CA 94025 USA; Department of Pathology, Oregon Health & Science University, 3181 SW Sam Jackson Park Road, Portland, OR 97239 USA; Department of Health Research and Policy, Stanford University School of Medicine, 900 Blake Wilbur Drive, Stanford, CA 94305 USA; Cancer Prevention Institute of California (CPIC), 2201 Walnut Avenue, Fremont, CA 94538 USA; Department of Psychiatry and The Dartmouth Institute for Health Policy and Clinical Practice, Geisel School of Medicine, 1 Rope Ferry Road, Lebanon, NH 03755 USA; Department of Pathology, Beth Israel Deaconess Medical Center and Harvard Medical School, 330 Brookline Avenue, Boston, MA 02215 USA

## Abstract

**Introduction:**

Screening mammography has contributed to a significant increase in the diagnosis of ductal carcinoma *in situ* (DCIS), raising concerns about overdiagnosis and overtreatment. Building on prior observations from lineage evolution analysis, we examined whether measuring genomic features of DCIS would predict association with invasive breast carcinoma (IBC). The long-term goal is to enhance standard clinicopathologic measures of low- versus high-risk DCIS and to enable risk-appropriate treatment.

**Methods:**

We studied three common chromosomal copy number alterations (CNA) in IBC and designed fluorescence *in situ* hybridization-based assay to measure copy number at these loci in DCIS samples. Clinicopathologic data were extracted from the electronic medical records of Stanford Cancer Institute and linked to demographic data from the population-based California Cancer Registry; results were integrated with data from tissue microarrays of specimens containing DCIS that did not develop IBC versus DCIS with concurrent IBC. Multivariable logistic regression analysis was performed to describe associations of CNAs with these two groups of DCIS.

**Results:**

We examined 271 patients with DCIS (120 that did not develop IBC and 151 with concurrent IBC) for the presence of 1q, 8q24 and 11q13 copy number gains. Compared to DCIS-only patients, patients with concurrent IBC had higher frequencies of CNAs in their DCIS samples. On multivariable analysis with conventional clinicopathologic features, the copy number gains were significantly associated with concurrent IBC. The state of two of the three copy number gains in DCIS was associated with a risk of IBC that was 9.07 times that of no copy number gains, and the presence of gains at all three genomic loci in DCIS was associated with a more than 17-fold risk (*P* = 0.0013).

**Conclusions:**

CNAs have the potential to improve the identification of high-risk DCIS, defined by presence of concurrent IBC. Expanding and validating this approach in both additional cross-sectional and longitudinal cohorts may enable improved risk stratification and risk-appropriate treatment in DCIS.

**Electronic supplementary material:**

The online version of this article (doi:10.1186/s13058-015-0623-y) contains supplementary material, which is available to authorized users.

## Introduction

Screening mammography is responsible for most diagnoses of asymptomatic ductal carcinoma *in situ* (DCIS) [1–3], raising concern for overtreatment of this nonlethal disease. In contrast to invasive breast carcinoma (IBC), radiation therapy (RT) has not demonstrated a survival benefit for DCIS [4], yet clinical trial subset analyses have failed to identify a patient subgroup that derives no recurrence-free survival (RFS) benefit; similarly, we cannot identify which DCIS patients benefit from adjuvant endocrine therapy [5–7]. Understanding how DCIS evolves to IBC, in terms of genomic progression and temporal progression, may provide insight into addressing these screening issues.

We and others have previously performed genome-wide sequencing studies on progression of breast neoplasia, from hyperplasia to carcinoma *in situ* to invasive carcinoma. These studies indicate that there is a gradual somatic gain of copy number alterations (CNAs) and single nucleotide variations (SNVs) [8–12]. Our studies have examined hyperplasia, DCIS and IBC from cross-sectional samples, by both targeted sequencing [12] and whole genome sequencing [10], to identify genomic changes that occur in progression from these pathologically defined neoplasias. These data have identified specific genomic changes to pathologic lesions defined by morphology whose risks have previously been studied at an epidemiologic level [13], including common CNAs and SNVs that have been identified in IBC [14, 15]. These gradual genomic changes provide an opportunity to predict which DCIS lesions are likely to be associated with progression to IBC.

It is well recognized that risk stratifying DCIS is challenging because of its clinical and biological heterogeneity. An additional problem when considering genetic relationships (lineage analysis) and generating genetic biomarkers of risk, is that the standard of surgical care for DCIS is that the entire lesion is removed. Thus, studies that examine the recurrence of DCIS or emergence of IBC are not likely to directly address the genetic relationships between DCIS and IBC that are essential to our understanding as to how cancer develops genetically. A cross-sectional study (examining concurrent DCIS and IBC) addresses this issue directly. The natural genetic relationships of concurrent DCIS and IBC are preserved and have not been altered by treatments. These cross-sectional samples provide a good way to test potential genetic biomarkers, such as somatic SNVs and CNAs, on a large cohort.

A number of studies have previously examined the risk of DCIS recurrence using protein expression markers [16]; however, DNA copy number changes are common in early genomic lesions and may serve as more robust biomarkers due to their insensitivity to intratumoral factors such as hypoxia. In this study, we examined the accumulation of CNAs as a biomarker for developing IBC in noninvasive neoplasia. We generated a theoretical analysis of SNV and CNA frequencies in DCIS through a simulation experiment based on IBC data from The Cancer Genome Atlas (TCGA) [14]. Since genomic change appears to correlate with progression [9], we aimed to study these changes in a large cohort at the level of the preinvasive DCIS lesion, and to characterize its association with clinical and demographic data [17, 18]. These findings may enable the development of molecular tools for DCIS risk stratification, which is an urgent clinical need.

## Methods

### Data resource environment and patient identification

All available cases with enough tissue for sampling were identified in the Department of Pathology at Stanford University Hospital (SUH) from 2000 to 2007 with the diagnosis of either DCIS and no development of IBC over a median follow-up of 9 years or DCIS with concurrent IBC present, based on per protocol assessment by SUH pathologists. Surgical samples with sufficient tissue were collected with Health Insurance Portability and Accountability Act (HIPAA)-compliant Stanford University Institutional Review Board (IRB) approval (Protocol number 19482 and 22825). Because archival tissue was used, a waiver of consent was obtained. All research was approved by SUH and the State of California IRB (for use of state cancer registry data).

### Clinical data extraction and data addition

Using Oncoshare, a multisource data resource for breast cancer outcomes research, we extracted clinical data from SUH electronic medical records (EMRs) (Epic Systems, Verona, WI, USA) and from a SUH warehouse for clinical data collected before Epic implementation in 2007, the Stanford Translational Research Integrated Database Environment (STRIDE), as previously published [17, 18]. We requested state cancer registry (California Cancer Registry, CCR) records for all patients with breast cancer treated at SUH from 2000 through 2011. CCR and EMR records were linked using names, social security numbers, medical record numbers, and birthdates. All personal identifying information was removed [18].

### Simulation analysis of SNV and CNA frequencies as predictors of invasive carcinoma in DCIS

We performed a simulation experiment to provide insight into the types of genomic alterations (in terms of both frequency and magnitude of association with IBC) that are most likely to be useful in a genomic predictor of IBC risk in DCIS. To construct a simulated genomic dataset, we based the sample size on the number of samples available in our study set (151 cases and 129 controls). We then created frequency-based classes of genomic alterations in DCIS and classes of differential frequencies between cases that progressed to IBC and controls that did not. We based our DCIS frequency classes on preliminary data for SNV/CNA frequencies in TCGA [14], as little is currently known about SNV/CNA frequencies in DCIS. We first created three frequency-based classes of genomic alterations in DCIS: low frequency (5 %), mid frequency (15 %), and high frequency (30 %), and four classes of differential frequencies between cases and controls: highly differential (alteration frequency is threefold higher in cases versus controls), moderately differential (alteration frequency is 1.5-fold higher in cases versus controls), low-level differential (alteration is 1.25-fold higher in cases versus controls), and nondifferential (alteration frequency is generated from the same distribution in cases and controls). Based on data for SNV/CNA frequencies in IBC in TCGA, we modeled 45 % (ten of 22) of our alterations as low frequency (this group is representative of low-frequency breast cancer alterations such as MLL3 mutation, PTEN mutation and GATA3 mutation), 27 % (six of 22) as moderate frequency (representative of moderate frequency breast cancer alterations such as 11q13 gain, 8q24 gain, ERBB2 gain and CDH1 mutation), and 27 % (six of 22) as high frequency (representative of common breast cancer alterations such as TP53 mutation, PIK3CA mutation, 1q gain, 8q gain, 16p gain, 20q gain, 16q deletion, 17p deletion, 8p deletion, and 22q deletion, among other common arm-level CNAs) in the simulated DCIS samples. We modeled nine of the 22 features (41 %) as deriving from distributions with differential frequency in the cases versus controls. These nine features were equally distributed across the nine possible permutations of frequency (low, moderate, high) and magnitude of case versus control differential (low, moderate, high).

For each of 2000 iterations, we first constructed simulated case and control data sets (as described above). We then used L1-regularized logistic regression to build a predictor and performed tenfold cross-validation to select the optimal value for the λ tuning parameter. For each of the 2000 iterations, we recorded the overall model performance (area under the curve (AUC) on held-out cases in cross-validation for the top-performing value of λ), the number of active features in the top-performing model, and the population-wide frequency (low frequency, moderate frequency, high frequency) and underlying distribution (nondifferential, low-level differential, moderately differential, highly differential) that gave rise to the active features.

### Patient population and samples

Patient surgical samples diagnosed at SUH between 2000 and 2007 were selected for the presence of DCIS and constructed into a tissue microarray (TMA, TA-239) based on a previously described protocol [19, 20]. The size of the DCIS was not obtained. In brief, two experienced breast pathologists (KJ and RW) reevaluated the grading for this study and the criteria used included architectural pattern and the presence of necrosis. Samples were excluded due to paucity of material or poor preservation of material. The TMA contained one representative 0.6 mm core from 280 clinically independent tumors, 151 samples of DCIS only, and 129 samples of DCIS with concurrent IBC. Sampling of DCIS in close proximity to, or intermixed with extensive invasive cancer was avoided. A total of 271 patients with DCIS only (120 cases) or DCIS and IBC (151 cases) were included in the final analysis. Note that there were seven cases that contributed two gene profiles and one case that contributed three gene profiles. For the primary analysis, we used all 280 samples. As a sensitivity analysis, we randomly selected one sample from each case that contributed more than one gene profile, and used a total of 271 samples corresponding to the 271 unique patients.

### Patient characteristics

In the 271 patients (280 samples) with DCIS, most were 40–64 years old and diagnosed from 2000 to 2003. Most (73.4 %) of patients were non-Hispanic (NH) white, with 19.6 % Asian/Pacific Islander, 3.7 % Hispanic, and 1.5 % NH black. Half (50.6 %) of the cases expressed hormone receptors (HR), and the most common grade was 2 (48 %). Among DCIS with IBC cases that had HR and human epidermal growth factor receptor 2 (HER2) status recorded, there was a roughly equivalent distribution between HR-positive HER2-negative (29.1 %), HER2-positive (35.8 %), and HR-negative, HER2-negative (triple-negative, 22.5 %) subtypes (Table 1 and see Additional file 1). HER2 gain was present in 30.8 % of the DCIS-only cases and 34.4 % of the DCIS with concurrent invasive cancer cases (Table 1). Treatments and outcomes varied somewhat by invasiveness: unilateral mastectomy was performed among 23.3 % DCIS-only and 31.1 % of DCIS with IBC patients, whereas bilateral mastectomy was performed among 20.8 % of DCIS-only and 27.8 % of DCIS with IBC patients. The rates of these surgical therapies are consistent with a study by Worni et al. where they found the rate of unilateral mastectomies in DCIS patients to be 23.4 % [21]. Only 8.3 % of DCIS-only patients were dead as of 2013, versus 19.9 % of DCIS with IBC patients (see Additional file 2).

**Table 1:** Characteristics of 271 patients with ductal carcinoma *in situ* (DCIS), with and without invasive breast cancer

			DCIS			
	All patients		DCIS only		DCIS with invasive cancer	
	N	(%)	N	(%)	N	(%)
Total	271	(100.0)	120	(100.0)	151	(100.0)
Age at diagnosis, years						
<40	24	(8.9)	4	(3.3)	20	(13.2)
40–49	90	(33.2)	43	(35.8)	47	(31.1)
50–64	90	(33.2)	36	(30.0)	54	(35.8)
≥65	67	(24.7)	37	(30.8)	30	(19.9)
Year of breast cancer diagnosis						
2000–2003	226	(83.4)	97	(80.8)	129	(85.4)
2004–2007	43	(15.9)	22	(18.3)	21	(13.9)
2008–2011	2	(0.7)	1	(0.8)	1	(0.7)
Race						
Missing	5	(1.8)	2	(1.7)	3	(2.0)
Hispanic	10	(3.7)	4	(3.3)	6	(4.0)
Non-Hispanic (NH) white	199	(73.4)	81	(67.5)	118	(78.1)
NH black	4	(1.5)	3	(2.5)	1	(0.7)
NH Asian/Pacific Islander	53	(19.6)	30	(25.0)	23	(15.2)
Socioeconomic status						
Missing	30	(11.1)	19	(15.8)	11	(7.3)
1 (Lowest)	4	(1.5)	2	(1.7)	2	(1.3)
2	15	(5.5)	8	(6.7)	7	(4.6)
3	23	(8.5)	9	(7.5)	14	(9.3)
4	40	(14.8)	10	(8.3)	30	(19.9)
5 (Highest)	159	(58.7)	72	(60.0)	87	(57.6)
Hormone receptors						
Missing	12	(4.4)	0	0	12	(7.9)
Either ER or PR positive	137	(50.6)	65	(54.2)	72	(47.7)
Both ER and PR negative	122	(45.0)	55	(45.8)	67	(44.4)
Stage						
Missing	4	(1.5)	0	0	4	(2.6)
Stage 0	120	(44.3)	120	(100.0)	0	0
Stage I	69	(25.5)	0	0	69	(45.7)
Stage II	69	(25.5)	0	0	69	(45.7)
Stage III	8	(3.0)	0	0	8	(5.3)
Stage IV	1	(0.4)	0	0	1	(0.7)
Grade						
Missing	2	(0.7)	1	(0.8)	1	(0.7)
1	56	(20.7)	27	(22.5)	29	(19.2)
2	130	(48.0)	57	(47.5)	73	(48.3)
3	83	(30.6)	35	(29.2)	48	(31.8)
HER2 gain						
No gain	182	(67.2)	83	(69.2)	99	(65.6)
Gain	89	(32.8)	37	(30.8)	52	(34.4)

*ER* estrogen receptor, *PR* progesterone receptor, *HER2* human epidermal growth factor receptor 2

### Fluorescence in situ hybridization

Fluorescence *in situ* hybridization (FISH) was performed to examine chromosome 1q32, 8q24 and 11q13 gains. The genomic loci targeted were chosen based on the simulation results (see Results) and their frequency in invasive cancers from The Cancer Genome Atlas (TCGA) data [14]. We used 4 μm formalin-fixed, paraffin-embedded sections cut from the constructed TMA, based on a protocol previously described [22]. Briefly, BAC clones RP11-1044H13 (1q32), RP11-1136L8 (8q24.21) and RP11-94L15 (17q12) were obtained from the BACPAC Resources Center (Children’s Hospital Oakland Research Institute, Oakland, CA, USA), while clone CTD-2537F6 (11q13.3) was acquired from Invitrogen/Life Technologies (Grand Island, NY, USA). Probe RP11-1044H13 (1q32), RP11-1136L8 (8q24.21) and CTD-2537F6 (11q13.3) were labeled with Cy3 dUTP (cat number PA53022 GE Healthcare, Pittsburgh, PA, USA) and control probes RP11-1120M18 (3q25) and CTD-2344F21 (2q37) were labeled with AlexaFluor 647-aha-dUTP (cat number A32763 Life Technologies) and Green dUTP (cat number 02N32-050 Abbot Molecular, Des Plaines, IL, USA), respectively using the Nick Translation Kit (cat number 07J00-001 Abbot Molecular).

### Scoring FISH

Imaging and analysis were performed using Ariol 3.4v software (Genetix/Leica Microsystems, San Jose, CA, USA). Fluorescence was scored visually using filters Cy3dUTP (green: 550 nm), AF 647 dUTP (red: 647 nm), and Green dUTP (yellow: 488 nm). Within the DCIS cells, total signals for each color within a given slide region were counted. Invasive carcinoma cells and nonneoplastic cells were excluded from the analysis. Signals from 100 cells per sample were counted, when possible, with a minimum of 40 cells counted in all cases. The test probes were individually hybridized with the two control probes for each genomic locus to determine copy number gain. Total test probe green counts (1q32, 8q24.21, 11q13.11 or 17q12) were compared with red (3q25) and yellow (2q37) control counts, which are frequently unaltered in breast cancer [14, 15]. The signals were scored according to two parameters; signals per cell and ratio of test probe to control probes. Only the DCIS components were scored and compared across cases, which were either DCIS alone or DCIS with concurrent IBC. Cases were scored as heterogeneous if at least 25 % of the scored DCIS cells had a different signal call. Cases were scored as gained if the target to control probe ratio was greater than 1.5 or the number of test signals was greater than three per cell. This scoring criterion was based on our previous study where we examined the HER2 copy number in a large cohort of breast cancers and a gain of greater than 1.5 was the cutoff value that most correlated with a worse outcome [23]. Cases were scored as deleted if the target to control probe ratio was less than 0.75, or greater than 25 % of the DCIS cells scored had a target to control probe ratio of less than 0.75. For consistency, we scored HER2 gain according to the criteria above set forth for the three genomic loci investigated.

### Immunohistochemistry

HER2 immunoreactivity was evaluated by immunohistochemistry (IHC). The TMA were cut into 4-μm-thick sections, deparaffinized, hydrated, subjected to Cell Conditioning 1 (CC1, Ventana Medical Systems, Tuscon, AZ, USA) antigen retrieval and stained with a prediluted anti-HER2 antibody (Rabbit, Clone 4B5, Ventana Medical Systems number 790-2991) using an automated immunostainer. HER2 expression was scored according to the 2013 American Society of Clinical Oncology/College of American Pathologists HER2 Test recommendations [24].

### Statistical analyses

We used logistic regression techniques to characterize the association between copy number gains at three loci and IBC among patients with DCIS. The multivariable model on which our primary analysis was based additionally included age at diagnosis, race, and hormone receptor status, grade of the DCIS component and HER2 gain in order to gauge the association of copy number status and IBC after adjusting for demographics and relevant clinical variables. A complete-case analysis was based on a model that included subjects who had data on all variables specified (N = 158). As a sensitivity analysis, we additionally employed multiple-imputation methods with ten imputed data sets (mi impute chained in Stata) to retain all subjects in the study even if they were missing one of the variables specified in the model (N = 271) (Stata Statistical Software. Release 13. StataCorp LP, College Station, TX, USA). A two-sided Wald test was conducted at the 0.05 level to assess the significance of the association. Odds ratios and 95 % confidence intervals were used to characterize the magnitude of the association. As a sensitivity analysis, we randomly selected one sample for cases with multiple gene profiles and repeated the logistic regression analyses with 154 cases in the complete case analysis and 271 cases in the analysis that employed multiple imputation.

## Results

### Simulation analysis of SNV and CNA frequencies as predictors of invasive carcinoma in DCIS

**Fig. 1:**
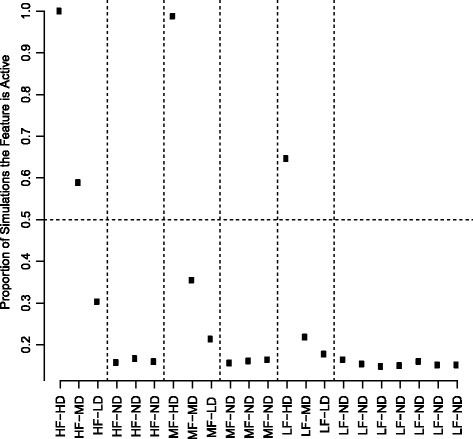
Genomic predictor simulation experiment. We created three frequency-based classes of genomic alterations in ductal carcinoma *in situ* (DCIS): low frequency (LF) (5 %), mid frequency (MF) (15 %), and high frequency (HF) (30 %), and four classes of differential frequencies between cases and controls: highly differential (HD) (alteration frequency is threefold higher in cases versus controls), moderately differential (MD) (alteration frequency is 1.5-fold higher in cases versus controls), low-level differential (LD) (alteration is 1.25-fold higher in cases versus controls), and nondifferential (ND) (alteration frequency is generated from the same distribution in cases and controls). For each of 2000 simulations, we used L1-regularized logistic regression to build a predictor and performed tenfold cross-validation to select the optimal value for the λ tuning parameter. For each simulation, we recorded which types of features in terms of frequency (LF, MF, HF) and differential status (ND, LD, MD, HD) were active in the model. The results are displayed in the figure, with the feature types along the X-axis and the proportion of simulations in which each feature type was active in the model along the Y-axis

We conducted a simulation experiment to determine the types of genomic characteristics (based on frequency and association with IBC) most likely to be useful features in a predictive model of DCIS risk in IBC. The results from this analysis suggest that genomic features with moderate-to-high overall frequency (15–30 %) and high differential frequency between cases versus controls (threefold) are likely to be selected as active features in the predictive model, while lower frequency alterations and alterations with weaker associations with IBC are much less likely to be informative in a risk-prediction model (Fig. 1). For example, the simulated genomic features with moderate-to-high frequency and strong association with IBC were selected in ≥ 99 % of the iterations, while simulated genomic features with strong association with IBC but low population frequency were selected in only 65 % of the models (Fig. 1). The frequency of recurrent genomic alterations in IBC varies greatly between SNVs and CNAs, with less than eight SNVs occurring at a frequency greater than 5 % while more than 30 CNAs occur at a frequency greater than 15 % [15].

Based on our modeling results, we expect that the majority of genomic features in a successful genomic classifier will be CNAs with fewer, if any, SNVs. As such, we decided to investigate the association between three of the most common and recurrent IBC-associated CNAs (gains of genomic regions of 1q, 8q24, and 11q13) and IBC risk in DCIS.

### Univariate exploratory analyses of chromosomal gains in DCIS with or without invasive cancer

**Fig. 2:**
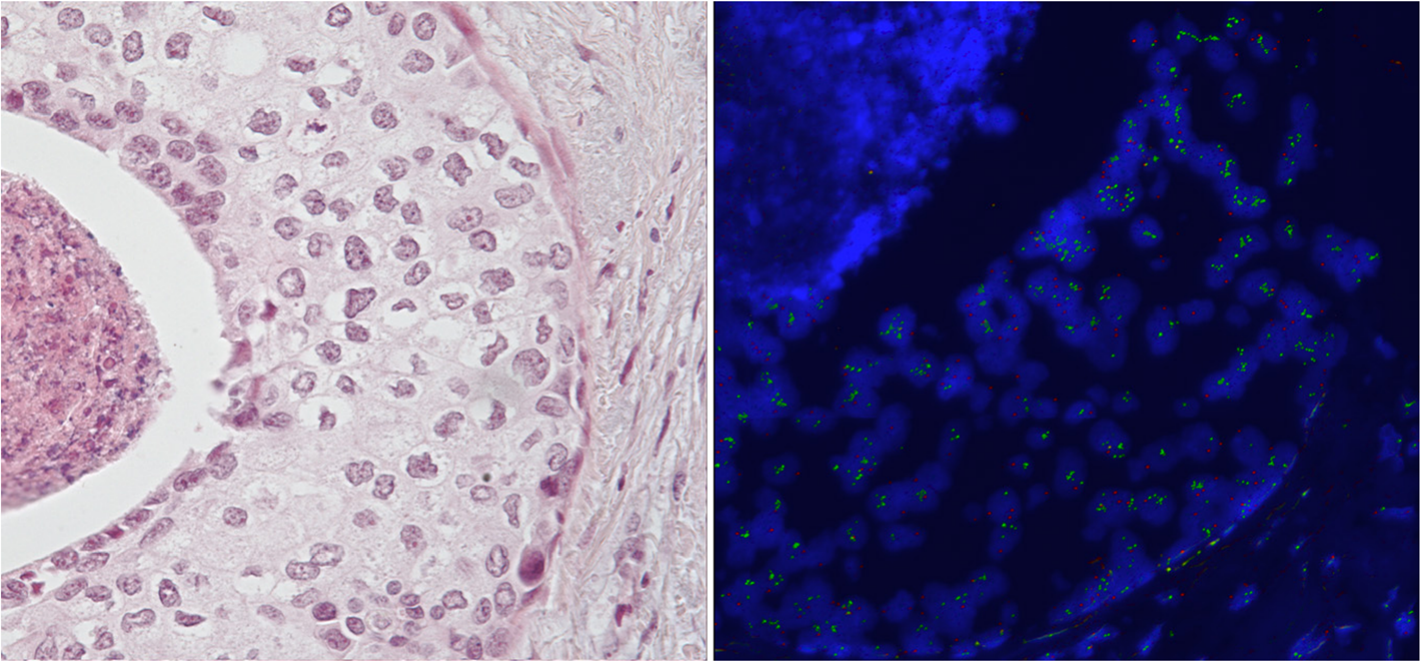
**a** Hematoxylin and eosin (H&E) image of ductal carcinoma *in situ* (DCIS) with 11q13 gain. **b** Fluorescence *in situ* hybridization (FISH) image of DCIS with high level of copy number gain

We examined the presence of copy number gains in three chromosomal loci, 1q, 8q24, and 11q13, by FISH in 280 samples diagnosed as DCIS only (122 cases with no development of IBC over a median follow-up period of 9 years), or DCIS plus IBC (158 cases) (Table 2) arrayed on a TMA. We chose to study a set of loci (1q, 8q24, and 11q13) which have a high frequency of copy number gains (>30 %) among at least two molecular breast cancer subtypes [14, 15]. The prevalence gains in all three genomic loci in the two groups of DCIS together were lower than values previously reported in IBC [15]. Overall copy number gain frequency was as follows: 1q at 52 % (compared to 64 % in IBC [15]), 8q24 at 44 % (compared to 60 % in IBC [15]), and 11q13 at 20 % (compared to 32 % in IBC [15]). Low copy number gains (one to two additional copies) represented the vast majority of copy number alterations at 1q and 8q24 (80 % and 78 %, respectively). In contrast, 11q13 had roughly equal numbers of low (53 %) and high (47 % with > 2 additional copies) gains (Fig. 2). When stratifying DCIS on whether there was concurrent IBC or not, we found increased genomic gains in DCIS with concurrent IBC (in comparison to DCIS alone) in all three regions when examined individually; in combinations; and with all three copy number gains. The prevalence of copy number gain was higher in DCIS with concurrent IBC versus DCIS alone across all three genomic loci individually (1.35- to 3-fold), in combinations, and with all three copy number gains (Table 2). We examined the co-existence of HER2 gain and the other three loci gains in both diagnostic groups. The overall copy number gain frequency of HER2 was 32.9 %. The prevalence of HER2 gain was higher in DCIS with concurrent IBC versus DCIS alone (Table 2).

**Table 2:** Chromosomal gains in ductal carcinoma *in situ* (DCIS) with and without invasive cancer

			DCIS type			
	All patients		DCIS only		DCIS with invasive cancer	
	N	(%)	N	(%)	N	(%)
Total	280	(100.0)	122	(100.0)	158	(100.0)
1q gains						
No signal	80	(28.6)	35	(28.7)	45	(28.5)
No	96	(34.3)	49	(40.2)	47	(29.7)
Yes	104	(37.1)	38	(31.1)	66	(41.8)
8q24 gains						
No signal	63	(22.5)	27	(22.1)	36	(22.8)
No	121	(43.2)	63	(51.6)	58	(36.7)
Yes	96	(34.3)	32	(26.2)	64	(40.5)
11q13 gains						
No signal	74	(26.4)	32	(26.2)	42	(26.6)
No	143	(51.1)	71	(58.2)	72	(45.6)
Yes	63	(22.5)	19	(15.6)	44	(27.8)
HER2 gains						
No	188	(67.1)	84	(68.9)	104	(65.8)
Yes	92	(32.9)	38	(31.1)	54	(34.2)
1q and 8q24 gains						
No signal	94	(33.6)	43	(35.2)	51	(32.3)
No	133	(47.5)	63	(51.6)	70	(44.3)
Yes	53	(18.9)	16	(13.1)	37	(23.4)
1q and 11q13 gains						
No signal	108	(38.6)	46	(37.7)	62	(39.2)
No	138	(49.3)	67	(54.9)	71	(44.9)
Yes	34	(12.1)	9	(7.4)	25	(15.8)
1q and HER2 gains						
No signal	80	(28.6)	35	(28.7)	45	(28.5)
No	145	(51.8)	66	(54.1)	79	(50.0)
Yes	55	(19.6)	21	(17.2)	34	(21.5)
8q24 and 11q13 gains						
No signal	87	(31.1)	37	(30.3)	50	(31.6)
No	159	(56.8)	78	(63.9)	81	(51.3)
Yes	34	(12.1)	7	(5.7)	27	(17.1)
8q24 and HER2 gains						
No signal	63	(22.5)	27	(22.1)	36	(22.8)
No	163	(58.2)	74	(60.7)	89	(56.3)
Yes	54	(19.3)	21	(17.2)	33	(20.9)
11q13 and HER2 gains						
No signal	74	(26.4)	32	(26.2)	42	(26.6)
No	165	(58.9)	78	(63.9)	87	(55.1)
Yes	41	(14.6)	12	(9.8)	29	(18.4)
1q and 8q24 and 11q13 gains						
No signal	112	(40.0)	49	(40.2)	63	(39.9)
No	146	(52.1)	68	(55.7)	78	(49.4)
Yes	22	(7.9)	5	(4.1)	17	(10.8)
1q and 8q24 and HER2 gains						
No signal	94	(33.6)	43	(35.2)	51	(32.3)
No	150	(53.6)	66	(54.1)	84	(53.2)
Yes	36	(12.9)	13	(10.7)	23	(14.6)
1q and 11q13 and HER2 gains						
No signal	105	(37.5)	44	(36.1)	61	(38.6)
No	140	(50.0)	68	(55.7)	72	(45.6)
Yes	35	(12.5)	10	(8.2)	25	(15.8)
8q24 and 11q13 and HER2 gains						
No signal	113	(40.4)	44	(36.1)	69	(43.7)
No	167	(59.6)	78	(63.9)	89	(56.3)
All four gains						
No signal	112	(40.0)	49	(40.2)	63	(39.9)
No	148	(52.9)	68	(55.7)	80	(50.6)
Yes	20	(7.1)	5	(4.1)	15	(9.5)
Mutually exclusive categories						
Unable to determine	112	(40.0)	49	(40.2)	63	(39.9)
No gains	43	(15.4)	29	(23.8)	14	(8.9)
1q only	31	(11.1)	15	(12.3)	16	(10.1)
8q24 only	19	(6.8)	8	(6.6)	11	(7.0)
11q13 only	8	(2.9)	4	(3.3)	4	(2.5)
Two of three gains	45	(16.1)	12	(9.8)	33	(20.9)
All three gains	22	(7.9)	5	(4.1)	17	(10.8)

After finding the chromosomal gains of 1q, 8q24 and 11q13 to be increased in DCIS in the setting of IBC compared to DCIS only, we tested whether these gains are associated with IBC (Table 3 and see Additional file 3). We found statistically significant differences in distribution of copy number gains between the two diagnostic groups in all three regions when examined individually, in combination, and with all three copy number gains. The sensitivity for each of the three regions alone ranged from 37.9 to 58.4 %, with high specificity for the combinations of gains of 1q and 11q13 (88.2 %); 8q24 and 11q13 (91.8 %); and all three copy number gains (93.2 %). The combination of 8q24 and 11q13 gains demonstrated the highest positive predictive values at 79.4 %. When we examined the co-existence of copy number gains of HER2 and the other three genomic loci, we found a statistically significant difference in the frequency distribution between the two diagnostic groups for the cytogenetic combination of 1q, 11q13 and HER2 (*p* = 0.038, Table 3). The sensitivity for this combination performed at the low end when compared to the three copy number gain combinations at 25.8 %, with a specificity of 87.2 % and a negative predictive value of 48.6 % (Table 3).

**Table 3:** Performance of cytogenetic combinations as predictors of invasive breast cancer

Chromosomal region gains	1q	8q24	11q13	HER2	1q and 8q24	1q and 11q13	1q and HER2	8q24 and 11q13	8q24 and HER2	11q13 and HER2	1q and 8q24 and 11q13	All four
All cases	200.0	217.0	206.0	280.0	186.0	172.0	200.0	193.0	217.0	206.0	168.0	168.0
DCIS plus invasive cancer (IBC)	113.0	122.0	116.0	158.0	107.0	96.0	113.0	108.0	122.0	116.0	95.0	95.0
DCIS only (no IBC)	87.0	95.0	90.0	122.0	79.0	76.0	87.0	85.0	95.0	90.0	73.0	73.0
True positive	66.0	64.0	44.0	54.0	37.0	25.0	34.0	27.0	33.0	29.0	17.0	15.0
False negative	47.0	58.0	72.0	104.0	70.0	71.0	79.0	81.0	89.0	87.0	78.0	80.0
False positive	38.0	32.0	19.0	38.0	16.0	9.0	21.0	7.0	21.0	12.0	5.0	5.0
True negative	49.0	63.0	71.0	84.0	63.0	67.0	66.0	78.0	74.0	78.0	68.0	68.0
Sensitivity	58.407	52.459	37.931	34.177	34.579	26.042	30.088	25.0	27.049	25.000	17.895	15.789
Specificity	56.322	66.316	78.889	68.852	79.747	88.158	75.862	91.765	77.895	86.667	93.151	93.151
Positive predictive value	63.462	66.667	69.841	58.696	69.811	73.529	61.818	79.412	61.111	70.732	77.273	75.000
Negative predictive value	51.042	52.066	49.650	44.681	47.368	48.551	45.517	49.057	45.399	47.273	46.575	45.946
Fisher’s exact test, two-tailed	0.046	0.006	0.010	0.610	0.034	0.022	0.425	0.002	0.432	0.052	0.040	0.094

*DCIS* ductal carcinoma *in situ*, *IBC* invasive breast cancer

### Multivariable logistic regression and classifier analyses predicting invasive cancer among DCIS cases

To characterize the association between DCIS and IBC, we applied multivariable models of IBC as a function of a six-level categorical variable describing chromosomal gains at regions 1q, 8q24 and 11q13, along with age at diagnosis, race, hormone receptor status, histological grade and the presence of HER2 gain (Table 4 and see Additional file 4). The association between copy number gain and IBC was statistically significant in both complete-case analysis and multiple-imputation (MI) analysis (*p* = 0.0013, 0.0001, respectively) and shows that subjects with gains at all three loci are 18 times more likely to have an IBC diagnosis than subjects without gains at these loci; subjects with exactly two copy number gains are nine times more likely to have an IBC diagnosis, and subjects with 8q24 gain only are 4.2 times more likely to have IBC than subjects with no gain in these regions (MI analysis). Interestingly, the genomic copy number gain, age at diagnosis and HER2 gain were the only statistically significant variables in the model. Of note, HER2 gain is not significantly associated with invasive cancer in the univariate analysis, but is inversely associated in the multivariate analysis, in which subjects with HER2 copy number gain were significantly less likely to have an IBC diagnosis (odds ratio 0.47, *p* = 0.039), when compared to DCIS alone (Table 4). In addition, we examined HER2 “high” amplification (defined as > 10 copies per nucleus) and HER2 strong positivity (defined as 3+ IHC staining) and neither of these variables was significantly associated with invasive cancer on either univariate or multivariate analyses.

**Table 4:** Univariate and multivariable logistic regression analyses predicting invasive breast cancer among ductal carcinoma *in situ* (DCIS) cases

	Univariate		Multivariable (complete cases = 158)		Multiple imputation (all cases = 280)	
	OR (95 % CI)	*P* value	OR (95 % CI)	*P* value	OR (95 % CI)	*P* value
Gene category		0.0027		0.0013		0.0001
No gain	1.00		1.00		1.00	
1q only	2.21 (0.85, 5.71)		1.96 (0.68, 5.67)		4.60 (1.77, 11.91)	
8q24 only	2.85 (0.94, 8.66)		4.23 (1.15, 15.50)		4.24 (1.27, 14.11)	
11q13 only	2.07 (0.45, 9.52)		2.51 (0.42, 15.00)		3.17 (0.65, 15.47)	
Two of three gains	5.70 (2.27, 14.27)		9.07 (2.93, 28.11)		7.91 (2.79, 22.41)	
All three gains	7.04 (2.16, 23.00)		17.96 (3.92, 82.24)		14.23 (3.56, 57.79)	
Age at diagnosis, years		0.015		0.0527		0.0253
<40	3.38 (1.07, 10.64)		3.60 (0.74, 17.50)		4.60 (1.28, 16.49)	
40–49	0.69 (0.38, 1.23)		0.46 (0.19, 1.12)		0.71 (0.37, 1.38)	
50–64	1.00		1.00		1.00	
≥65	0.57 (0.31, 1.08)		0.70 (0.26, 1.89)		0.68 (0.33, 1.39)	
Race		0.092		0.1478		0.3085
Non-Hispanic (NH) white	1.34 (0.50, 3.61)		3.99 (0.70, 22.70)		1.56 (0.51, 4.76)	
NH Asian/Pacific Islander	0.68 (0.23, 2.04)		2.10 (0.31, 14.23)		0.91 (0.25, 3.29)	
Other	1.00		1.00		1.00	
Hormone receptors		0.4896		0.6824		0.4258
ER and/or PR positive	1.00		1.00		1.00	
Both ER and PR negative	1.19 (0.73, 1.92)		1.18 (0.53, 2.61)		1.26 (0.71, 2.25)	
Grade		0.9263		0.8397		0.5300
1	1.00		1.00		1.00	
2	1.13 (0.61, 2.08)		0.93 (0.36, 2.40)		0.73 (0.36, 1.52)	
3	1.12 (0.57, 2.18)		0.73 (0.23, 2.29)		0.62 (0.26, 1.46)	
HER2 any gain		0.5926		0.0102		0.0386
No	1.00		1.00		1.00	
Yes	1.15 (0.69, 1.90)		0.29 (0.12, 0.75)		0.47 (0.23, 0.96)	

*ER* estrogen receptor, *PR* progesterone receptor

## Discussion

This study demonstrates that genomic changes can act as a risk stratifier for DCIS, predicting the presence of concurrent IBC. We observed no significant differences between DCIS patients with and without concurrent IBC in standard clinicopathologic factors of race, hormone receptor status and histological grade. By contrast, we did find significantly higher frequencies for copy number gains at 1q, 8q24 and 11q13 with any two of three genomic loci and all three genomic loci in patients with DCIS and concurrent invasive cancer when compared to DCIS only. Multivariable analysis showed that gains at the three regions were significantly associated with IBC among patients with DCIS, after adjustment for important clinical variables including grade, hormone receptor status and even HER2 copy number gain, which was associated with a lower risk of having invasive cancer and is consistent with prior publications on DCIS [25]. Furthermore, we show that this is a feasible method, utilizing standardized FISH techniques, and as such has high potential to address the critical unmet need for accurate risk stratification and personalized treatment of DCIS.

Population-wide screening mammography has largely created the problem of diagnosing asymptomatic DCIS [1–3]; concerns about overtreatment have lent support for replacing “DCIS” with “ductal intraepithelial neoplasia”, emphasizing the indolent behavior of many of these lesions [26]. However, since we cannot predict which DCIS lesions will progress to invasive cancer, treatment guidelines recommend mastectomy or breast-conserving therapy plus RT, followed by adjuvant tamoxifen: this approach is excessive treatment for most patients [7, 27–30]. Previous attempts at risk stratification, using protein expression markers such as p16, Ki67 and COX [16], or an RT-PCR assay that estimates the risk of local recurrence [31], are limited by problems of intratumoral variability and reliance upon IBC rather than DCIS for gene selection. Some genetic changes occur early in tumorigenesis and therefore are likely present in most of the neoplastic population at more advanced stages like DCIS [10]. DNA copy number changes are common in early genomic lesions and may be more robust as biomarkers than gene expression levels, which can be subject to heterogeneity due to intratumoral factors such as hypoxia. At the molecular level, CNAs and SNVs have been described previously in breast cancer [14, 15], and their application and integration into clinical practice is appealing. Our present modeling results show that CNAs are more likely to be prognostic than SNVs based on their frequency in IBC [15].

Our approach aimed to optimize practicality for ultimate translation to patient care. We used TMA technology because the amount of DCIS in each core is similar to the amount present in conventional breast biopsies. We used FISH to measure CNAs as this approach can generate single-cell measurements in a complex tumor microenvironment with multiple cell types present. Although molecular techniques are sensitive for detection and quantification at the SNV level [32], critical morphological correlation is lost. Our use of FISH on TMAs avoids this limitation, resulting in more precise genomic copy number data. The FISH technique is also currently used in the clinics to measure HER2 in breast cancer (and other more subtle genomic alterations in other neoplasia) and thus this approach may be easily adopted by most clinical laboratories.

Our cross-sectional study approach has limitations and advantages over a longitudinal approach. While a cross-sectional study does not allow for the evaluation of recurrence, we have a median follow-up of 9 years for the DCIS-only cases, a timeframe consistent with previous studies examining the recurrence rates of DCIS [33]. While the challenges of clinical biomarker assessment will ultimately be addressed with longitudinal cohorts of DCIS that progress over time to IBC, longitudinal cohorts do not address the genetic relationships between DCIS and IBC that are essential to our understanding as to how cancer develops. The problem with longitudinal cohorts is that the initial DCIS should be entirely removed at the time of the definitive surgical treatment. Therefore, the resulting subsequent recurrence, either DCIS or IBC, is likely not directly related to the primary DCIS. Given that the surgical treatment of the primary DCIS is to entirely remove the DCIS but not necessarily remove potentially related lesions of lesser risk (e.g., hyperplasias), a longitudinal DCIS cohort study would be more reflective of the risk potential of the associated lesser risk lesions that are not entirely removed. Alternatively, the recurrence may be directly related to the primary DCIS if the surgical resection is incomplete. However, genetic biomarkers generated from this scenario would not be related to intrinsic features of the primary DCIS but rather more complex treatment effects such as the clinical and radiologic appreciation of the extent of the disease. It is also possible that a clonally related neoplastic precursor, such as atypical ductal hyperplasia or columnar cell change, is present at the surgical margin and that residual part of this lesion progresses to the recurrent carcinoma. This would explain the observation that the recurrences typically occur in the same quadrant of the breast. This scenario is compatible with our lineage evolutionary tree analyses as determined by whole genome sequencing, where we can identify precursors in both columnar cell lesions and atypical ductal hyperplasia that are clonally related to both the concurrent ductal carcinoma *in situ* and the invasive carcinoma [10]. It is also possible that there is a nonneoplastic field effect, localized to that quadrant that is responsible for the recurrence. Additional studies on the lineages of the initial and recurrent lesions will be required to understand this fully. The main limitation of this cross-sectional approach is that it does not address the important clinical scenario of whether a patient with DCIS alone will eventually develop IBC. This is clearly an important question to address. However, as noted above, this question is less about the intrinsic features of DCIS than about the features of the neoplasia (e.g., hyperplasia) that remains unresected at the time of definitive surgery. Prior to tackling that question, it is useful to identify, on an evolutionary level, whether genomic changes within DCIS and its evolutionary ancestors predict the development of IBC. From this perspective of identifying features in DCIS that predict risk, a cross-sectional study is appropriate as the natural evolutionary relationships between DCIS and IBC are retained.

Although the FISH assay we developed did identify high-risk DCIS cases, there are multiple subtypes of IBC and likely multiple corresponding subtypes of DCIS, and as such different combinations of markers may be needed for risk stratification of different DCIS subtypes. Our understanding of the different pathways involved in the development of IBC and specific genomic alterations therein is growing. Low- and high-grade neoplasias demonstrate different CNAs [34]. In addition, *PIK3CA* mutation occurs early in oncogenesis and is associated with ductal hyperplasias, while *TP53* mutations at early stages have not been found [35, 12]. Furthermore, NOTCH/MAST fusions have been described in cases of DCIS associated with IBC [36]. This growing knowledge will serve to guide future studies of the approach we present here.

## Conclusions

In conclusion, our proof-of-principle study demonstrates the feasibility of a novel genomic predictor of breast cancer risk using data derived from TCGA, and characterizes its performance in the context of patient demographic and clinical factors. The three FISH assays for 1q, 8q24 and 11q13 positively identified a subset of high-risk DCIS patients; if expanded and validated in prospective trials, this approach, which can be integrated into routine clinical practice readily, may ultimately improve the care of patients with early breast neoplasia.

## Additional files

Additional file 1:
**Tumor marker subtype of patients with ductal carcinoma** ***in situ*** **(DCIS), with and without invasive breast cancer.** (DOC 31 kb) Additional file 2:
**Treatments and outcomes of patients with ductal carcinoma** ***in situ*** **(DCIS), with and without invasive breast cancer.** (DOC 32 kb) Additional file 3:
**Performance of cytogenetic combinations as predictors of invasive cancer.** (DOC 55 kb) Additional file 4:
**Univariate and multivariable logistic regression analyses predicting invasive cancer among ductal carcinoma** ***in situ*** **(DCIS) cases: sensitivity analysis using one randomly selected observation per patient.** *ER* estrogen receptor, *PR* progesterone receptor. (DOC 58 kb)

## Abbreviations

AUC: area under the curve
CCR: California Cancer Registry
CNA: copy number alterations
DCIS: ductal carcinoma *in situ*
EMR: electronic medical record
ER: estrogen receptor
FISH: fluorescence *in situ* hybridization
H&E: hematoxylin and eosin
HER2: human epidermal growth factor receptor 2
HIPAA: Health Insurance Portability and Accountability Act
HR: hormone receptors
IBC: invasive breast cancer
IHC: immunohistochemistry
IRB: Institutional Review Board
NH: non-Hispanic
PR: progesterone receptor
RFC: recurrence-free survival
RT: radiation therapy
SNV: single nucleotide variation
STRIDE: Stanford Translational Research Integrated Database Environment
SUH: Stanford University Hospital
TCGA: The Cancer Genome Atlas
TMA: tissue microarray
