# Supplementary material for: Chromosomal copy number alterations for associations of ductal carcinoma in situ with invasive breast cancer
Source: Breast Cancer Res. 2015 Aug 13;17(1):108. doi: 10.1186/s13058-015-0623-y (PMC4534146; doi:10.1186/s13058-015-0623-y)
Supplement: Additional file 1: — Tumor marker subtype of patients with ductal carcinoma in situ (DCIS), with and without invasive breast cancer. (DOC 31 kb) [file 13058_2015_623_MOESM1_ESM.doc]

Additional file 1. Tumor marker subtype of 271 patients with ductal carcinoma in situ (DCIS), with and without invasive breast cancer.

|  |  | | ***DCIS*** | | | |
| --- | --- | --- | --- | --- | --- | --- |
|  | *All Patients* | | *DCIS only* | | *DCIS with invasive cancer* | |
|  | *N* | *(%)* | *N* | *(%)* | *N* | *(%)* |
| ***Tumor marker subtype*** |  |  |  |  |  |  |
| *Missing* | 19 | (7.0) | 0 | 0 | 19 | (12.6) |
| *HR+, HER2-* | 44 | (16.2) | 0 | 0 | 44 | (29.1) |
| *HER2+* | 54 | (19.9) | 0 | 0 | 54 | (35.8) |
| *Triple-* | 34 | (12.5) | 0 | 0 | 34 | (22.5) |
| *N/A (Stage 0)* | 120 | (44.3) | 120 | (100.0) | 0 | 0 |
