# Supplementary material for: Chromosomal copy number alterations for associations of ductal carcinoma in situ with invasive breast cancer
Source: Breast Cancer Res. 2015 Aug 13;17(1):108. doi: 10.1186/s13058-015-0623-y (PMC4534146; doi:10.1186/s13058-015-0623-y)
Supplement: Additional file 2: — Treatments and outcomes of patients with ductal carcinoma in situ (DCIS), with and without invasive breast cancer. (DOC 32 kb) [file 13058_2015_623_MOESM2_ESM.doc]

Additional file 2. Treatments and outcomes of patients with ductal carcinoma in situ (DCIS), with and without invasive breast cancer.

|  |  | | ***DCIS*** | | | |
| --- | --- | --- | --- | --- | --- | --- |
|  | *All Patients* | | *DCIS only* | | *DCIS with Invasive Breast Cancer* | |
|  | *N* | *(%)* | *N* | *(%)* | *N* | *(%)* |
| ***Total*** | 271 | (100.0) | 120 | (100.0) | 151 | (100.0) |
| *Mastectomy, Unilateral* | 75 | (27.7) | 28 | (23.3) | 47 | (31.1) |
| *Mastectomy, Bilateral* | 67 | (24.7) | 25 | (20.8) | 42 | (27.8) |
| *Post-Mastectomy Radiation Treatment* | 18 | (6.6) | 0 | 0 | 18 | (11.9) |
| *Post-Lumpectomy Radiation Treatment* | 79 | (29.2) | 37 | (30.8) | 42 | (27.8) |
| *Chemotherapy* | 71 | (26.2) | 0 | 0 | 71 | (47.0) |
| *Death* | 40 | (14.8) | 10 | (8.3) | 30 | (19.9) |
