# Supplementary material for: Chromosomal copy number alterations for associations of ductal carcinoma in situ with invasive breast cancer
Source: Breast Cancer Res. 2015 Aug 13;17(1):108. doi: 10.1186/s13058-015-0623-y (PMC4534146; doi:10.1186/s13058-015-0623-y)
Supplement: Additional file 3: — Performance of cytogenetic combinations as predictors of invasive cancer. (DOC 55 kb) [file 13058_2015_623_MOESM3_ESM.doc]

Additional file 3. Performance of cytogenetic combinations as predictors of invasive cancer.

| *Chromosomal region gains* | *1q* | *8q24* | *11q13* | *HER2* | *1q and 8q24* | *1q and 11q13* | *1q and HER2* |
| --- | --- | --- | --- | --- | --- | --- | --- |
| All cases | 200.0 | 217.0 | 206.0 | 280.0 | 186.0 | 172.0 | 200.0 |
| DCIS plus invasive cancer (IBC) | 113.0 | 122.0 | 116.0 | 158.0 | 107.0 | 96.0 | 113.0 |
| DCIS only (no IBC) | 87.0 | 95.0 | 90.0 | 122.0 | 79.0 | 76.0 | 87.0 |
| True positive | 66.0 | 64.0 | 44.0 | 54.0 | 37.0 | 25.0 | 34.0 |
| False negative | 47.0 | 58.0 | 72.0 | 104.0 | 70.0 | 71.0 | 79.0 |
| False positive | 38.0 | 32.0 | 19.0 | 38.0 | 16.0 | 9.0 | 21.0 |
| True negative | 49.0 | 63.0 | 71.0 | 84.0 | 63.0 | 67.0 | 66.0 |
| Sensitivity | 58.407 | 52.459 | 37.931 | 34.177 | 34.579 | 26.042 | 30.088 |
| Specificity | 56.322 | 66.316 | 78.889 | 68.852 | 79.747 | 88.158 | 75.862 |
| Positive predictive value | 63.462 | 66.667 | 69.841 | 58.696 | 69.811 | 73.529 | 61.818 |
| Negative predictive value | 51.042 | 52.066 | 49.650 | 44.681 | 47.368 | 48.551 | 45.517 |
| Fisher's exact test, 2 tailed | 0.046 | 0.006 | 0.010 | 0.610 | 0.034 | 0.022 | 0.425 |

| *Chromosomal region gains* | *8q24 and 11q13* | *8q24 and HER2* | *11q13 and HER2* | *1q and 8q24 and 11q13* | *1q and 8q24 and HER2* | *1q and 11q13 and HER2* | *8q24 and 11q13 and HER2* | *All 4* |
| --- | --- | --- | --- | --- | --- | --- | --- | --- |
| All cases | 193.0 | 217.0 | 206.0 | 168.0 | 186.0 | 175.0 | 167.0 | 168.0 |
| DCIS plus invasive cancer (IBC) | 108.0 | 122.0 | 116.0 | 95.0 | 107.0 | 97.0 | 89.0 | 95.0 |
| DCIS only (no IBC) | 85.0 | 95.0 | 90.0 | 73.0 | 79.0 | 78.0 | 78.0 | 73.0 |
| True positive | 27.0 | 33.0 | 29.0 | 17.0 | 23.0 | 25.0 |  | 15.0 |
| False negative | 81.0 | 89.0 | 87.0 | 78.0 | 84.0 | 72.0 | 89.0 | 80.0 |
| False positive | 7.0 | 21.0 | 12.0 | 5.0 | 13.0 | 10.0 |  | 5.0 |
| True negative | 78.0 | 74.0 | 78.0 | 68.0 | 66.0 | 68.0 | 78.0 | 68.0 |
| Sensitivity | 25.0 | 27.049 | 25.000 | 17.895 | 21.495 | 25.773 |  | 15.789 |
| Specificity | 91.765 | 77.895 | 86.667 | 93.151 | 83.544 | 87.179 | 100.000 | 93.151 |
| Positive predictive value | 79.412 | 61.111 | 70.732 | 77.273 | 63.889 | 71.429 |  | 75.000 |
| Negative predictive value | 49.057 | 45.399 | 47.273 | 46.575 | 44.000 | 48.571 | 46.707 | 45.946 |
| Fisher's exact test, 2 tailed | 0.002 | 0.432 | 0.052 | 0.040 | 0.455 | 0.038 | 0.038 | 0.094 |
