# Supplementary material for: Chromosomal copy number alterations for associations of ductal carcinoma in situ with invasive breast cancer
Source: Breast Cancer Res. 2015 Aug 13;17(1):108. doi: 10.1186/s13058-015-0623-y (PMC4534146; doi:10.1186/s13058-015-0623-y)
Supplement: Additional file 4: — Univariate and multivariable logistic regression analyses predicting invasive cancer among ductal carcinoma in situ (DCIS) cases: sensitivity analysis using one randomly selected observation per patient. ER estrogen receptor, PR progesterone receptor. (DOC 58 kb) [file 13058_2015_623_MOESM4_ESM.doc]

| Additional file 4. Univariate and multivariable logistic regression analyses predicting invasive cancer among ductal carcinoma in situ (DCIS) cases: sensitivity analysis using one randomly selected observation per patient. ER, estrogen receptor; PR, progesterone receptor. | | | | | | |
| --- | --- | --- | --- | --- | --- | --- |
|  | *Univariate* | | *Multivariable (Complete cases = 158)* | | *Multiple Imputation (All cases = 280)* | |
|  | *OR (95%CI)* | *P-value* | *OR (95%CI)* | *P-value* | *OR (95%CI)* | *P-value* |
| ***Gene Category*** |  | 0.0026 |  | 0.0082 |  | 0.0109 |
| *No gains* | 1.00 |  | 1.00 |  | 1.00 |  |
| *1q only* | 2.07 (0.79, 5.40) |  | 1.46 (0.52, 4.16) |  | 2.56 (1.03, 6.40) |  |
| *8q24 only* | 2.96 (0.93, 9.42) |  | 2.56 (0.74, 8.85) |  | 2.65 (0.80, 8.77) |  |
| *11q13 only* | 2.07 (0.45, 9.52) |  | 1.90 (0.35, 10.33) |  | 2.12 (0.45, 10.00) |  |
| *Two of three gains* | 6.03 (2.36, 15.37) |  | 5.19 (1.91, 14.1) |  | 4.86 (1.85, 12.78) |  |
| *All three gains* | 6.63 (2.02, 21.78) |  | 7.09 (1.93, 26.10) |  | 6.00 (1.72 (20.86) |  |
| ***Age at Diagnosis, years*** |  | 0.014 |  | 0.1196 |  | 0.0322 |
| *<40* | 3.33 (1.05, 10.56) |  | 3.45 (0.72, 16.49) |  | 4.66 (1.33, 16.27) |  |
| *40-49* | 0.73 (0.40, 1.32) |  | 0.56 (0.24, 1.33) |  | 0.83 (0.44, 1.57) |  |
| *50-64* | 1.00 |  | 1.00 |  | 1.00 |  |
| *65* | 0.54, 0.28, 1.03) |  | 0.91 (0.35, 2.39) |  | 0.69 (0.35, 1.39) |  |
| ***Race*** |  | 0.1102 |  | 0.1559 |  | 0.1345 |
| *Non Hispanic (NH) White* | 1.46 (0.49, 4.31) |  | 3.43 (0.52, 22.47) |  | 1.76 (0.55, 5.69) |  |
| *NH Asian/Pacific Islander* | 0.77 (0.24, 2.50) |  | 1.66 (0.22, 12.61) |  | 0.91 (0.25, 3.29) |  |
| *Other* | 1.00 |  | 1.00 |  | 1.00 |  |
| ***Hormone Receptors*** |  | 0.6198 |  | 0.7671 |  | 0.8035 |
| *ER and/or PR-positive* | 1.00 |  | 1.00 |  | 1.00 |  |
| *Both ER and PR-negative* | 1.13 (0.69, 1.85) |  | 0.89 (0.41, 1.95) |  | 1.08 (0.61, 1.91) |  |
| ***Grade*** |  | 0.7748 |  | 0.8637 |  | 0.7232 |
| *1* | 1.00 |  | 1.00 |  | 1.00 |  |
| *2* | 1.19 (0.64, 2.24) |  | 0.88 (0.34, 2.24) |  | 0.81 (0.39, 1.66) |  |
| *3* | 0.28 (0.65, 2.52) |  | 0.73 (0.23, 2.30) |  | 0.71 (0.31, 1.64) |  |
